# *DiasMorph*: a dataset of morphological traits and images of Central European diaspores

**DOI:** 10.1038/s41597-024-03607-3

**Published:** 2024-07-16

**Authors:** Roberta L. C. Dayrell, Lina Begemann, Tankred Ott, Peter Poschlod

**Affiliations:** 1 Faculty of Biology and Preclinical Medicine, University of Regensburg, Universitätsstraße 31, Regensburg, D-93053 Germany; 2 Royal Botanic Gardens, Kew, Wakehurst, Ardingly, Haywards Heath, West Sussex RH17 6TN UK

**Keywords:** Plant reproduction, Databases

## Abstract

We present *DiasMorph*, a dataset of images and traits of diaspores from 1,442 taxa in 519 genera, and 96 families from Central Europe, totalling 94,214 records. The dataset was constructed following a standardised and reproducible image analysis method. The image dataset consists of diaspores against a high-contrast background, enabling a simple and efficient segmentation process. The quantitative traits records go beyond traditional morphometric measurements, and include colour and contour features, which are made available for the first time in a large dataset. These measurements correspond to individual diaspores, an input currently unavailable in traits databases, and allow for several approaches to explore the morphological traits of these species. Additionally, information regarding the presence and absence of appendages and structures both in the images and diaspores of the assessed taxa is also included. By making these data available, we aim to encourage initiatives to advance on new tools for diaspore identification, further our understanding of morphological traits functions, and provide means for the continuous development of image analyses applications.

## Background & Summary

The morphological description of seeds and diaspores offers essential information for scientists and practitioners in a wide variety of fields, including botany, restoration, conservation, ethnobotany, archaeology, and agriculture. Diaspore traits, such as size, shape, colour, surface structures, and the presence of appendages are needed to establish the identity of particular diaspores that become detached of their mother plant^1^, for instance in seed lots, seed traps, soil seed bank, archaeological sites, or forensic investigations. Moreover, integrating diaspore morphological traits into theoretical plant regeneration frameworks can lead to major advances in predictive evolutionary and ecological models, and thereby support conservation and restoration actions^2^.

Throughout the years, the demand for knowledge of diaspore morphology has led to numerous compilations of text descriptions and/or images of diaspores in books, guides and atlases^1,3–7^. In the last two decades, databases have been built to synthesise and centralise information on diaspore traits, facilitating large scale analyses^8–11^. Along with databases, standardised protocols were established for trait measurements to allow for the integration of data with different sources. These included methods for the description of diaspores, which consist of the quantification of size and other morphometric measurements (most reported as taxa mean or range values), and the classification of attributes either based on visual (perceptual) categories and/or functional structures and/or anatomical parts^12^.

Recently, the pressing need for new solutions to deal with environmental crises, together with the surge in applications of machine learning and image analysis in ecology and related fields, calls for an upgrade of the diaspore morphological datasets. The automated extraction of information from digital images provides the opportunity to collect quantitative phenotypic data in large quantities, enabling the investigation of high dimensional and complex relationships between traits and their interaction with environmental variables^13^. Furthermore, the use of machine learning algorithms to classify images and/or suites of traits can allow for the automation of taxa identification, making the task faster and not exclusively dependent on experienced taxonomists^14,15^.

Here, we present *DiasMorph*, a comprehensive dataset of morphological traits and images of diaspores from Central Europe. It provides images of 94,214 diaspores from 1,442 taxa in 519 genera, and 96 families, captured against a high-contrast background with a standardised and reproducible method^16^. The dataset also compiles information on quantitative morphological traits extracted from the images following an image analysis method^16^ and include not only traditional morphometric measurements, but also colour, and contour features made available for the first time in a large dataset. The quantitative traits records correspond to measurements of individual diaspores, an input currently unavailable in trait databases that will allow for several approaches to be used for a complete exploration of the morphological traits of these species. We also included information on the presence and absence of appendages and structures both in the diaspores and images of the evaluated taxa. By making these data available, we aim to encourage initiatives to advance on new tools for diaspore identification, further our understanding of morphological traits functions, enhance existing databases, and provide means for the continuous development of image analyses applications.

## Methods

**Fig. 1:**
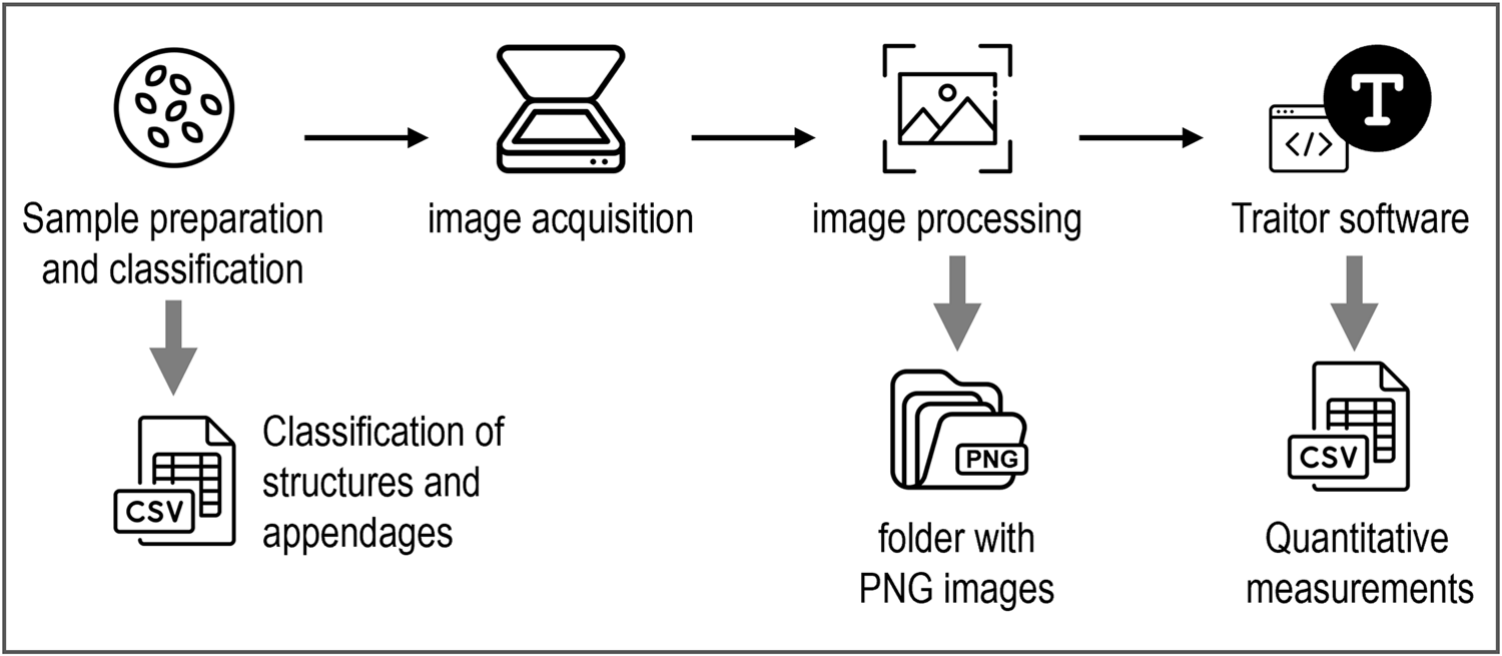
Workflow overview for dataset.

The workflow for seed trait extraction consists of sample preparation, qualitative traits assessment, image acquisition, image processing and trait measurement with Traitor software (Fig. 1).

### Sampled taxa

**Fig. 2:**
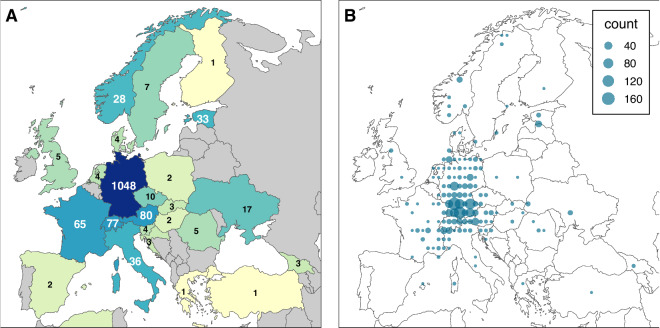
Maps showing the number of diaspore collections (**A**) per country (**B**) per locality or geometric centre in the *DiasMorph* dataset. In (**B**), coordinates are rounded and grouped to the nearest whole degree. To enhance visualisation, four countries (Ethiopia, Iceland, India, and Namibia), each with a single collection, have been omitted.

We sampled diaspores available in the seed collection of the Chair of Ecology and Conservation Biology at the University of Regensburg, Germany, which was started and curated by Prof. Peter Poschlod. The collection comprises taxa found in Central Europe, with collections carried out mainly in Germany (Fig. 2), and serves as a reference for identifying diaspores collected during field studies in the region. While Germany is home to 4,202 taxa^17^ (species and infraspecific taxa) of seed plants, the collection includes 1,048 taxa sourced from Germany, representing about 25% of the country’s flora, making it a substantial and representative sample. Most diaspores were collected within Central Europe, ensuring regional relevance. Additionally, some taxa with wide global distributions that encompass Central Europe were sourced from other areas, further enhancing the dataset’s comprehensiveness.

**Fig. 3:**
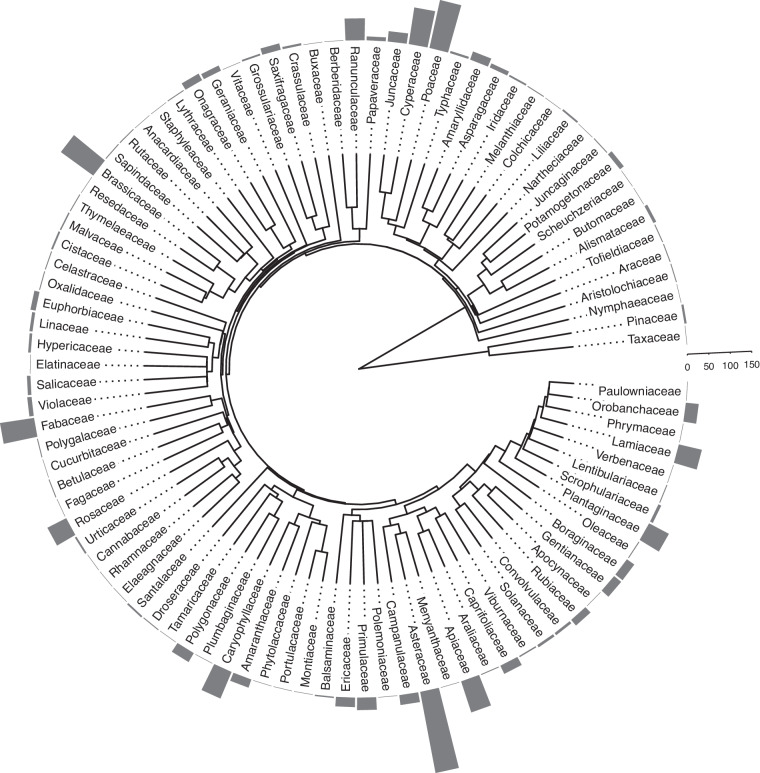
Cladogram of the phylogeny for the families in the *DiasMorph* dataset. The barplot represents the number of taxa within each family in the *DiasMorph* dataset.

In total, our dataset contains images and records of quantitative morphological traits for 94,214 diaspores from 1,442 taxa (including species, infraspecific taxa, and three sections), belonging to 519 genera, 96 plant families (Fig. 3). Taxon names and family information were checked and updated using the functions *WFO.match and WFO.one* from the R package *WorldFlora*^18^. The last nomenclature verification was carried out on May 20^th^, 2024. The most represented families in the database are Asteraceae (192 taxa; 65 genera), Poaceae (114; 48), Brassicaceae (93; 44), Cyperaceae (86; 10), and Fabaceae (80; 22). This distribution closely reflects the diversity of the most species-rich families within the region^17^. However, there is an exception: the Rosaceae family is underrepresented due to limited collections of the genus *Rubus*, which comprises hundreds of taxa.

### Geolocation

Since coordinates were not readily available for the diaspore collection, we utilised Google Maps to approximate the coordinates for each location. Subsequently, we categorised each location based on its resolution: locality (1,036 cases), which involved specific places such as neighbourhoods, towns, villages, parks, cities, mountain peaks, and communes; region (136 cases), encompassing larger areas such as districts and states within countries; country (50 cases); mountain range (156 cases); river (69 cases); botanic garden (9 cases); and commercial supplier (1 case). The obtained coordinates represent the geometric centre of a polyline (e.g., a river) or polygon (e.g., a region).

### Recorded appendages

**Fig. 4:**
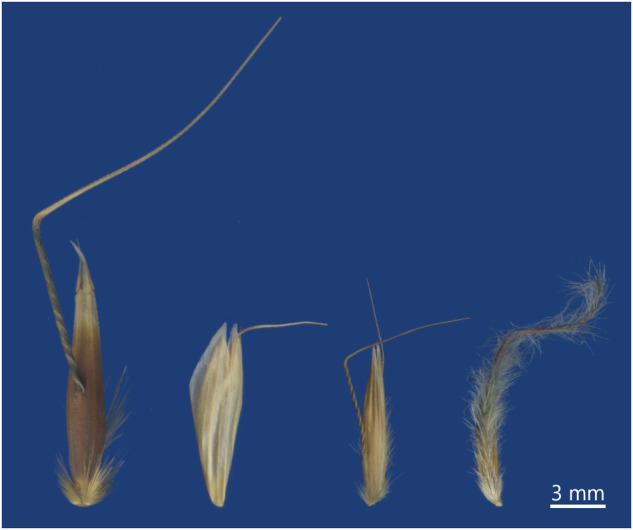
Example of taxa classified as having bent elongated appendages (first three from left to right) or bearing distinctively crooked elongated appendages (rightmost). From left to right: *Avena barbata, Bromus squarrosus, Arrhenatherum elatius* (Poaceae)*, Pulsatilla alpina* (Ranunculaceae).

For each species, we recorded diaspore structures and appendages (Table 1, Fig. 4) following a modified version of seed structure categories in LEDA Trait standards^8,12^. As LEDA is a database focused on functional traits, the modifications aimed to improve the objectivity of the classification and facilitate the recognition of morphological structures for identification purposes. For each taxon, appendages and structures were classified as present (1) or absent (0). In some instances, diaspores of species and genera were found with and without appendages and structures; for these cases, we recorded the structures as present and later specified them as missing from the image (*see* Sample Preparation).

**Table 1:** Summary of the diaspore appendage and structure categories.

Appendage/Structure	Description
1. Fleshy cover	Fleshy or cup-like structure enveloping the seeds.
2. Fleshy appendage	Fleshy structure attached to seed.
3. Dry covering structure	Dry covering structures that partially or completely cover the germination unit.
4. Flat appendage	Flat, delicate structures protruding from the germination unit.
5. Hairy appendage	Tuft, hairlike branches, or ring of hairs/scales attached to the germination unit.
6. Elongated appendages	Structures that stick out of the diaspore, having a length considerably greater than width, and thickness.
6.1 Short	Between one tenth and half of the diaspore’s length.
6.2 Long	More than half of the diaspore’s length.
6.3 Spiral coiled	Spiral coiled elongated appendage.
6.4 Bent	Bent elongated appendage (Fig. 4).
6.5 Hairy	Appendage covered with fine, flexible outgrowths.
6.6 Bristles	Linear, semiflexible outgrowths.
6.7 Multiple	Two or more elongated appendages.
7.1 Single hook	One hook-like appendage.
7.2 Multiple hooks	Bristles or spines with curved or backwards pointing tips.
8.1 Surface hairs	Fine, flexible, linear outgrowths on the surface.
8.2 Surface bristles	Semiflexible, thicker bristles on the surface.

This table provides a concise overview of the various types of diaspore appendages and structures, including their key characteristics. A more detailed version of this table, with extensive descriptions and additional information, is available as supplementary material (Supplementary Table 1).

### Extraction of quantitative traits

We used an image analysis method described and validated by Dayrell *et al*.^16^ to obtain images and extract quantitative measurements of diaspore morphology.

#### Sample preparation

We cleaned the diaspores with the aid of a stereo microscope and only selected diaspores with all structures in a well-preserved state, apart from three exceptions. (1) Fleshy covering structures and some fleshy outgrowths were removed due to the pronounced changes that these structures undergo after dispersal, which can lead to unrecognisable colours, shapes, and sizes. (2) We measured diaspores without scales or covering structures when most diaspores in a vial of the seed collection had detached from these structures without handling. (3) Hairy appendages (e.g., pappus and plumes) were removed due to requirements of the method^16^. The structures that were not present in the scanned diaspores were recorded as ‘missing structures’ in the dataset.

#### Image acquisition

**Fig. 5:**
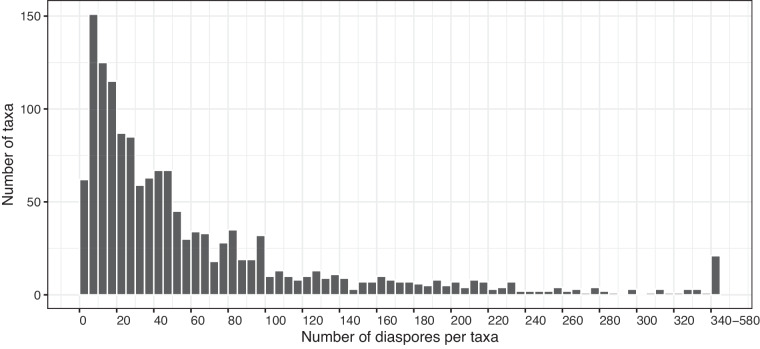
Histogram of the number of diaspores per taxon sampled for quantitative measurements.

For image acquisition, diaspores were arranged on the flat scanner avoiding any contact or overlap. The number of sampled diaspores varied for each taxon according to their availability in the seed collection (Fig. 5). We sampled all available material that met sample preparation standards when 30 or fewer diaspores were available. In cases where the number of available diaspores exceeded 30, we sampled seeds to cover an area of up to 200 cm^2^. The flatbed scanner was covered with a wooden frame 10 mm thick with a royal blue background. Images were acquired with a flatbed scanner (HP Scanjet G4010) at a resolution of 1,200 DPI to well-represent small seeds and fine appendages. All automatic correction functions associated with the scanner software were disabled to ensure that the RGB values of the samples were not manipulated. The resulting images were saved in the Joint Photographic Experts Group (JPEG) format with no compression.

#### Image processing

**Fig. 6:**
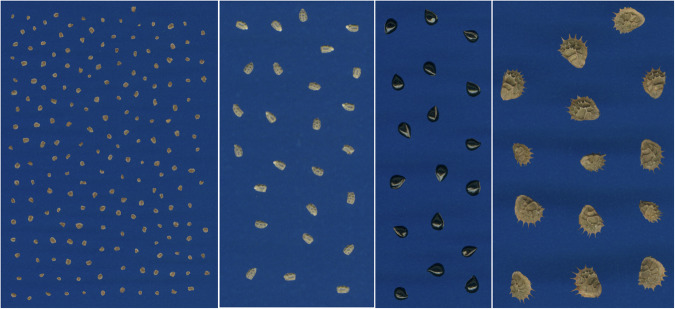
Examples of diaspore images in the *DiasMorph* dataset.

To allow standardisation of colour measurements, a Spyder Checkr^®^ 24 card (Datacolor, NJ, USA) was scanned in the flatbed scanner under the same settings as the diaspores, and used to calculate a colour conversion matrix (CCM). The CCM was then applied to images for optimal colour reproduction (https://github.com/rdayrell/colour_calibration). In some images, undesired elements, such as broken seeds and particles, were removed from image with the brush and clone stamp tools in Adobe Photoshop. Images were saved in PNG format throughout all processing steps to avoid compression artifacts. Processed images (Fig. 6) comprise the original image dataset and were used as inputs for automated trait extraction.

#### Extraction with traitor software

**Fig. 7:**
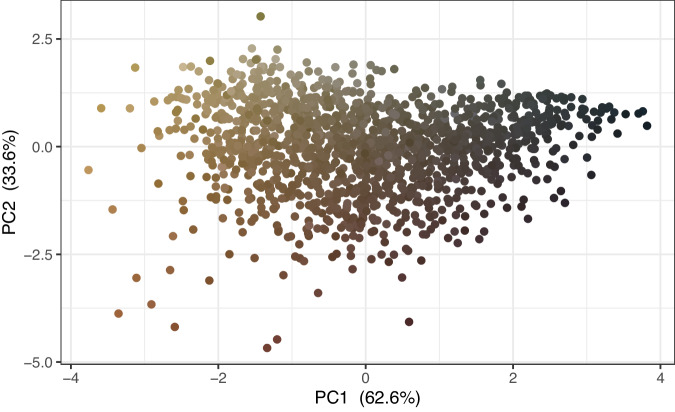
PCA scores plot obtained from the median colour values of diaspores from the taxa in the *DiasMorph* dataset.

The Traitor software https://github.com/TankredO/traitor was used to segment, align, and extract morphological traits from images^16^. The extracted traits include: (1) morphometric measurements (length, width, aspect ratio, area, perimeter, diaspore surface structure, solidity, circularity); (2) colour measurements for human recognition purposes (Fig. 7; mean, median, and most dominant colours in sRGB), and ecological and evolutionary studies (independent of any particular animal visual system; linear sRGB); (3) standardised contour of diaspores (50 coordinates for each seed) for shape analysis methods. After the extraction, fields containing size measurements in pixels were converted to units of measurement considering the conversion factor of 47.8 pixels per millimetres obtained from a reference scale, which is also included as an image in the dataset.

#### Algorithm limitation and correction

One limitation of the image-based trait extraction algorithm is its occasional failure to accurately align diaspores with bent elongated appendages (e.g. bent awns or distinctively crooked elongated appendages; Fig. 4), resulting in incorrect size and morphometric measurements^16^. Upon checking the consistency of Traitor’s output (see ‘Technical Validation’ section), this occurred primarily to taxa that belonged to Poaceae family, except for one Ranunculaceae species. Thus, the records of taxa with elongated bent or distinctively crooked appendages were deleted from the quantitative traits’ dataset obtained from original images, detailed in the previous section.

To provide reliable measurements of taxa with such appendages, we edited the original images of diaspores to make them compatible with the algorithm. We also edited images of Poaceae taxa bearing unbent elongated appendages, even though they provided correct outputs. This was done to provide measurements pertaining to the same structures, making the data consistent and comparable across all the Poaceae taxa. As a result of this correction process, the final quantitative dataset has two records for each diaspore of taxa with elongated unbent appendages, obtained from original and edited images, while there is only one record for each diaspore of taxa with elongated bent appendages, obtained from edited images.

Image editing consisted in manually erasing the elongated appendage from the image with the brush and clone stamp tools in Adobe Photoshop and saving the image as PNG. The edited images were labelled with the same name as the original image, with the addition of ‘_edit’ (e.g., ‘img_0261’ and ‘img_0261_edit’) and are available in a separate zip file. Traits of edited images were extracted with Traitor and merged with the quantitative dataset described in the previous section. For these images, ‘elongated appendages’ were classified as ‘missing structures’.

## Data Records

The authors of this study have publicly released *DiasMorph* dataset^19^, which is available at 10.6084/m9.figshare.21206507.v5.

Image dataset files and information

- Description for files in *DiasMorph*_original_images.zip**:** The zip file contains 1,547 colour images of 1,442 taxa in PNG format and 1,200 DPI resolution (10.48 GB).
- Description for files in *DiasMorph*_edited_images.zip**:** The zip file **(**226.4 MB) contains 41 colour images of 34 taxa in PNG format and 1,200 DPI resolution.
- Description for files in sample_images.zip**:** The zip file **(**36.57 MB) contains four sample images included in ‘*DiasMorph*_original_images.zip’.
- Description for file scale_cal.png: The png file (17.3 MB) provides a reference scale obtained with the same equipment and settings as the diaspore images.
  Tabular datasets files and information
- Description for dataset *DiasMorph*_labels_and_structures.csv: The dataset (197 KB) is coded with UTF-8 (allowing for the inclusion of German characters) and contains image labels and the recorded diaspore structures and appendages (Supplementary Table 2).

- Description for dataset *DiasMorph*_quantitative_traits.csv: The dataset (214.2 MB) contains image labels and the quantitative traits extracted from images (Supplementary Table 3).
- Description for text document *DiasMorph*_metadata.odt: The open text document (17 KB) contains Supplementary Tables 1–3.

## Technical Validation

The reliability of our compilation was assessed using the same datasets and validation method as in Dayrell *et al*.^16^. The only difference was how we converted Traitor measurements from pixels to millimetres: we used a conversion factor obtained from a scale, instead of relying on DPI values. For this, we used images of seeds from 1,228 taxa, which represents approximately 85% of the taxa in the *DiasMorph* dataset. We compared the average length and width values obtained by Traitor with the average manual measurements taken from seeds within the same collection^16^.The correlation between the measurements obtained through two different approaches was evaluated using Lin’s concordance correlation coefficient (ρc), a measure indicating the consistency of a new measurement with a standard one, ranging from 1 for perfect agreement to −1 for complete disagreement^20^. This assessment was performed utilising the ‘CCC’ function within the ‘DescTools’ package^21^.

The ρc for length and width measurements were 0.978 (95% CI [0.975–0.980]) and 0.983 ([0.981–0.985]), respectively. These results are very similar to those reported by Dayrell *et al*.^16^ and indicate a strong agreement between the image-based trait extraction and manual measurements. Additionally, Dayrell *et al*.^16^ manually inspected outlines and alignment outputs of measurements that exhibited less than 95% agreement between the two methods and found no issues with Traitor’s outputs and no systematic error.

## Usage Notes

The *DiasMorph* dataset offers images and standardised quantitative and qualitative data for individual diaspores from over 1,400 taxa found in Central Europe. As the dataset was constructed using a standardised and accessible approach, it is feasible to include new records to improve the representation of taxa and regions for comparisons, as well as to add structures like fleshy ones that have been preserved but are not currently included. Although considerable effort went into enhancing diaspore characterisation to provide objective measurements that are comparable across taxa, the dataset does not encompass the full diversity of morphological traits across all taxa. For instance, seed thickness (also referred to as ‘seed height’) is not included, and future solutions employing 3D scanners could help add this additional dimension to diaspore characterisation. Additionally, since the availability of diaspores per taxon varied within the collection, researchers conducting deep learning tasks should be mindful of this class imbalance when analysing the data.

### Supplementary information

Supplementary material

## Data Availability

Codes used in this publication are available at GitHub and deposited at Zedono as follows: colour calibration—https://github.com/rdayrell/colour_calibration^22^; Traitor—https://github.com/TankredO/traitor^23^.
