# Supplementary material for: DiasMorph: a dataset of morphological traits and images of Central European diaspores
Source: Sci Data. 2024 Jul 16;11:781. doi: 10.1038/s41597-024-03607-3 (PMC11252285; doi:10.1038/s41597-024-03607-3)
Supplement: Supplementary file 1 — Supplementary material [file 41597_2024_3607_MOESM1_ESM.doc]

Metadata for Dayrell, RLC, Begemann L, Ott T, Poschlod P. DiasMorph: a dataset of morphological traits and images of Central European diaspores.

Content: Supplementary Tables 1, 2 and 3

**Supplementary Table 1.** Summary of the diaspore appendage and structure categories.

| Appendage/Structure | Description |
| --- | --- |
| 1. Fleshy cover | Fleshy structure (e.g., pulp), or cup-like structure (e.g., aril) that totally or partly envelops the seeds. |
| 2. Fleshy appendage | Fleshy appendage or structure (e.g., elaiosome) attached to seed. It can vary in form, size, and position between species. |
| 3. Dry covering structure | Dry covering structures that partially or completely cover the germination unit. Presence was only recorded for species/genera in which diaspores can be found both with and without these structures (e.g., glumes of Poaceae, utricle of *Carex*, bracteoles of *Atriplex*). |
| 4. Flat appendage | Membranous / delicate structures that stick out of the more compact part of the germination unit with a flat, thin form. |
| 5. Hairy appendage | A tuft, hairlike branches, or ring of hairs (and less often scales) which are attached to the germination unit and can be removed from it. |
| 6. Elongated appendages | Structures that prominently stick out of the main part of the diaspore, having a length considerably greater than width, and height. These were classified into short or long and according to specialisations. (This category excludes hairs and hooks, which have their own categories). |
| 6.1 Elongated short | Elongated appendage is at least one tenth of the length and shorter than half of the diaspore’s length. Therefore, surface structures such as papillae, glands, tubercles, beaks, and short projections are not considered to be elongated appendages. |
| 6.2 Elongated long | Elongated appendage is at least half of the length of the diaspore. |
| 6.3 Elongated spiral coiled | Spiral coiled elongated appendage. |
| 6.4 Elongated bent | Elongated appendage is bent (Fig. 4). |
| 6.5 Elongated hairy | Elongated appendage has fine, flexible, linear outgrowths. Different from hairy appendage as the hairs, as these are covering the surface of the appendage and cannot be easily detached from it. |
| 6.6 Elongated bristles | Elongated appendage has linear outgrowths that are semiflexible, thicker and generally shorter than hairs. |
| 6.7 Elongated multiple | Two or more elongated appendages are present. |
| 7.1 Single hook | One elongated appendage with very recurved beak in a hook-like shape. |
| 7.2 Multiple hooks | Bristles or spines with curved or backwards pointing tips, or with secondary bristles along their length. |
| 8.1 Surface hairs | Fine, flexible, linear outgrowths on the surface. |
| 8.2 Surface bristles | Linear outgrowths on the surface that are semiflexible, thicker and generally shorter than hairs. |

**Supplementary Table 2.** Variable names and descriptions for file *DiasMorph*_labels_and_structures.csv. Definition of categories are in Supplementary Table 1.

| Variables | Description |
| --- | --- |
| image_name | Image file name in image dataset |
| scientificName | Accepted species name. |
| scientificNameAuthorship | Author of the species accepted name. |
| spec.name.ORIG | Species scientific name in the data source. |
| genus | Taxonomic genus. |
| family | Taxonomic Family. |
| location | Location of diaspore collection as recorded in the data source. |
| lat | Approximate latitude of diaspore collection. |
| lon | Approximate longitude of diaspore collection. |
| location_type | The resolution of the coordinates (see ‘Location’ section for details). |
| location_reference | Additional reference for the coordinates of diaspore collection in case of ambiguity. |
| country | The country where the diaspores were collected. |
| collection_date | Date of diaspore collection as recorded in the data source. |
| missing_structures | Structures that are not present in the image.* |
| c1_fleshy_cover | Absence (0), presence (1). |
| c2_fleshy_appendage | Absence (0), presence (1). |
| c3_dry_covering_structure | Absence (0), presence (1). |
| c4_flat_appendage | Absence (0), presence (1). |
| c5_hairy_appendage | Absence (0), presence (1). |
| c6_1_elongated_short | Absence (0), presence (1). |
| c6_2_elongated_long | Absence (0), presence (1). |
| c6_3_elongated_spiral_coiled | Absence (0), presence (1). |
| c6_4_elongated_bent | Absence (0), presence (1). |
| c6_5_elongated_hairy | Absence (0), presence (1). |
| c6_6_elongated_bristles | Absence (0), presence (1). |
| c6_7_elongated_multiple | Absence (0), presence (1). |
| c7_1_single_hook | Absence (0), presence (1). |
| c7_2_multiple_hooks | Absence (0), presence (1). |
| c8_1_surface_hairs | Absence (0), presence (1). |
| c8_2_surface_bristles | Absence (0), presence (1). |
| img_dataset | ‘original’ for images in ‘*DiasMorph*_original_images.zip’ and ‘edited’ for images in ‘*DiasMorph*_edited_images.zip’. |

Notes: * In most cases, the appendage and structure classification in our dataset reflects structures present in the diaspores and depicted in the images. However, in some instances, existing structures and appendages are absent from the image. This occurred when: 1) some diaspores of certain taxa were found with appendages and structures but most without; 2) fleshy covering structures and some fleshy outgrowths were removed due to pronounced morphological changes; 3) hairy appendages (e.g., pappus and plumes) were removed due to method requirements; 4) images edited to remove elongated appendages. See details in sections ‘Sample preparation’ and 'Algorithm Limitation and Correction'. For such cases, we recorded the structures as present in the classification, to reflect the actual diaspore morphology, while specifying if/which structures are missing from the image.

**Supplementary Table 3.** Variable names and descriptions for file *DiasMorph*_quantitative_traits.csv.

| Variables | Description |
| --- | --- |
| sample_name | Name of the single diaspore image extracted by Traitor. Unique for each diaspore. |
| image_name | Image file name in image dataset. |
| scientificName | Accepted species name. |
| scientificNameAuthorship | Author of the species accepted name. |
| spec.name.ORIG | Species scientific name in the data source. |
| genus | Taxonomic genus. |
| family | Taxonomic Family. |
| missing_structures | Structures that are not present in the diaspore image.* |
| length | the longest dimension of the diaspore in millimetres (mm) |
| width | the widest axis perpendicular to the length axis in millimetres (mm) |
| aspect_ratio | Proportional relationship between width and length. Dimensionless.  Formula: aspect ratio = width ÷ length. |
| area | Area of diaspore contour in square millimetres (mm^2^). |
| perimeter | Perimeter of diaspore contour in millimetres (mm). |
| surface_structure | Shape descriptor that describes the roughness of a diaspore's surface. The maximum value is 1 (completely smooth), and surface is increasingly rougher as the value approaches 0. Dimensionless.  Formula: surface structure = perimeter (convex hull) ÷ perimeter. |
| solidity | Shape descriptor that describes the overall concavity of a diaspore. The maximum value is 1 (completely convex shape), and lower values indicate greater extent of concavity and/or irregular boundaries. Dimensionless.  Formula: solidity = area (convex hull) ÷ area. |
| circularity | Shape descriptor that describes the degree of similarity to a perfect circle. The maximum value is 1 (perfect circle), and the shape is increasingly less circular as the value approaches 0.  Formula: circularity = 4π × area ÷ perimeter^2^. |
| R_median | Linearised median pixel value for the red (R) channel within the sRGB colour space. Range 0 (absence of channel) to 255 (maximum value). |
| G_median | Linearised median pixel value for the green (G) channel within the sRGB colour space. Range 0 (absence of channel) to 255 (maximum value). |
| B_median | Linearised median pixel value for the blue (B) channel within the sRGB colour space. Range 0 (absence of channel) to 255 (maximum value). |
| R_mean | Linearised mean pixel value for the red (R) channel within the sRGB colour space. Range 0 (absence of channel) to 255 (maximum value). |
| G_mean | Linearised mean pixel value for the green (G) channel within the sRGB colour space. Range 0 (absence of channel) to 255 (maximum value). |
| B_mean | Linearised mean pixel value for the blue (B) channel within the sRGB colour space. Range 0 (absence of channel) to 255 (maximum value). |
| sR_median | Median pixel value for the red (R) channel. sRGB colour space: range 0 (absence of channel) to 255 (maximum value). |
| sG_median | Median pixel value for the green (G) channel. sRGB colour space: range 0 (absence of channel) to 255 (maximum value). |
| sB_median | Median pixel value for the blue (B) channel. sRGB colour space: range 0 (absence of channel) to 255 (maximum value). |
| sR_mean | Mean pixel value for the red (R) channel. sRGB colour space: range 0 (absence of channel) to 255 (maximum value). |
| sG_mean | Mean pixel value for the green (G) channel. sRGB colour space: range 0 (absence of channel) to 255 (maximum value). |
| sB_mean | Mean pixel value for the blue (B) channel. sRGB colour space: range 0 (absence of channel) to 255 (maximum value). |
| rgb_0_r | Median pixel value for the red (R) channel of the second most dominant colour. sRGB colour space: range 0 (absence of channel) to 255 (maximum value). |
| rgb_0_g | Median pixel value for the green (G) channel of the second most dominant colour. sRGB colour space: range 0 (absence of channel) to 255 (maximum value). |
| rgb_0_b | Median pixel value for the blue (B) channel of the second most dominant colour. sRGB colour space: range 0 (absence of channel) to 255 (maximum value). |
| rgb_1_r | Median pixel value for the red (R) channel of the most dominant colour. sRGB colour space: range 0 (absence of channel) to 255 (maximum value). |
| rgb_1_g | Median pixel value for the green (G) channel of the most dominant colour. sRGB colour space: range 0 (absence of channel) to 255 (maximum value). |
| rgb_1_b | Median pixel value for the blue (B) channel of the most dominant colour. sRGB colour space: range 0 (absence of channel) to 255 (maximum value). |
| rgb_0_frac | Fraction of pixels assigned as the second most dominant colour. Range 0 to 0.5. |
| rgb_1_frac | Fraction of pixels assigned as the most dominant colour. Range 0.5 to 1. |
| x_0 – x_49 | x values for each of the 50 xy coordinates that comprise the diaspore contour after standardization for size invariance. Columns x_0 to x_49. |
| y_0 – y_49 | y values for each of the 50 xy coordinates that comprise the diaspore contour after standardization for size invariance. Columns y_0 to y_49. |

* See notes for Supplementary Table 2.
